# Taphonomic and technological analyses of Lower Palaeolithic bone tools from Clacton-on-Sea, UK

**DOI:** 10.1038/s41598-022-23989-x

**Published:** 2022-11-23

**Authors:** Simon A. Parfitt, Mark D. Lewis, Silvia M. Bello

**Affiliations:** 1 Institute of Archaeology, University College London, 31-34 Gordon Square, London, WC1H 0PY UK; 2 Centre for Human Evolution Research, Natural History Museum, Cromwell Road, London, SW7 5BD UK

**Keywords:** Archaeology, Anthropology

## Abstract

The exceptional survival of Middle Pleistocene wooden spears at Schöningen (Germany) and Clacton-on-Sea (UK) provides tantalizing evidence for the widespread use of organic raw materials by early humans. At Clacton, less well-known organic artefacts include modified bones that were identified by the Abbé Henri Breuil in the 1920s. Some of these pieces were described and figured by Hazzledine Warren in his classic 1951 paper on the flint industry from the Clacton Channel, but they have been either overlooked in subsequent studies or dismissed as the product of natural damage. We provide the first detailed analysis of two Clactonian bone tools found by Warren and a previously unrecognized example recovered in 1934 during excavations directed by Mary Leakey. Microscopic examination of percussion damage suggests the bones were used as knapping hammers to shape or resharpen flake tools. Early Palaeolithic bone tools are exceedingly rare, and the Clacton examples are the earliest known organic knapping hammers associated with a core-and-flake (Mode 1) lithic technology. The use of soft hammers for knapping challenges the consensus that Clactonian flintknapping was undertaken solely with hard hammerstones, thus removing a major technological and behavioural difference used to distinguish the Clactonian from late Acheulean handaxe (Mode 2) industries.

## Introduction

In his description of the Lower Palaeolithic Clactonian flint industry from Clacton-on-Sea, Essex (UK), Samuel Hazzledine Warren illustrates three bones [ref.^1^, Plate 6–7] that he regarded as having been worked and used. These potential ~ 400,000-year-old tools were illustrated but not described in detail; they were interpreted as a polished radius of a red deer, an axe-edged tool made from a rhinoceros radius and a ‘bar-hammer’ (billet) made from a deer tibia. Warren [ref.^1^, p.129] also refers to a second ‘bar-hammer’ made from a rhinoceros radius. The specimens have since fallen into obscurity and the consensus has followed Wymer’s [ref.^2^, p.282] opinion that these bones were modified by natural agencies. As a result, the Clacton ‘bar-hammers’ have been overlooked in reviews of the Clactonian^3–8^.

Although the oldest bone tools are found at Olduvai^9,10^ as early as 2.1 million years ago (Ma) and slightly later between 1 and 2 Ma at sites in southern Africa^11–13^, it isn’t until the later part of the Middle Pleistocene that bone tools become more widespread with examples from sites in the Near East and Europe. Recent studies^14–25^ have shown that Middle Pleistocene hominins used a variety of antler and bone implements as hammers and anvils to shape and re-sharpen flint tools. The adoption of soft hammers is a significant technological advance that allowed greater precision and refinement in lithic tool production usually associated with later Acheulean handaxes (Mode 2) and Levallois and Mousterian flake tools (Mode 3). The development of soft hammer knapping techniques is generally seen as reflecting increased cognitive abilities in Pleistocene hominin populations^26,27^, which separated these groups from hominins making technologically simpler (Mode 1) stone tools. This dichotomy is challenged, however, by the discovery of exceptionally well-preserved bone tools associated with a core-and-flake industry at the 300,000-year-old horse butchery and spear site at Schöningen, Germany^28^, which also includes the earliest known multipurpose bone tools^17,18^. Here, bones were used in various percussion tasks that involved anvils, knapping flint and cracking bones for their marrow^17,18,23,24^. These finds have prompted debates on the ‘level’ of cognitive ability and behavioural sophistication of early hominin populations and how bone tool technologies were maintained and transferred between different populations or independently acquired in separate geographical regions^22^.

These publications prompted our reassessment of the Clacton specimens. A further impetus for this study was the recent discovery (in the collections curated at the Natural History Museum, London, UK) of a metapodial hammer from Mary Leakey’s 1934 excavation of the Clacton Channel deposits at Jaywick Sands^29^.

In this paper, we document the macroscopic characteristics of the Clacton specimens, their microscopic features as revealed by 3D microscopy, and we interpret how these tools were used, based on comparisons with taphonomic datasets and modern experiments. We identify the Clacton bone tools as hammers that were used in the production and re-sharpening of flake tools; as such, they represent the earliest known examples of soft knapping hammers associated with a core-and-flake (Mode 1) lithic industry. As well as providing a new perspective on the technical and behavioural abilities of Clactonian hominins, the use of soft hammers removes an important distinction that was used to separate Clactonian tool production from the more refined late Acheulean handaxe industries^7^.

## The Clacton Channel and the Clactonian

The term ‘Clactonian’ was proposed by Warren^1^ to refer to a Lower Palaeolithic core-and-flake (Mode 1) industry from an ancient channel of the Thames-Medway at Clacton-on-Sea in eastern England. Subsequently, Clactonian artefacts were found at Swanscombe, Kent^30,31^, at Barnham, Suffolk^32,33^ and recently in a tributary of the Thames at Ebbsfleet^34,35^. These sites date to the early part of the Hoxnian Interglacial (Marine Isotope Stage (MIS) 11, 424–374 kya) and were replaced by a handaxe industry (Mode 2) about 415 kya^33^.

**Figure 1:**
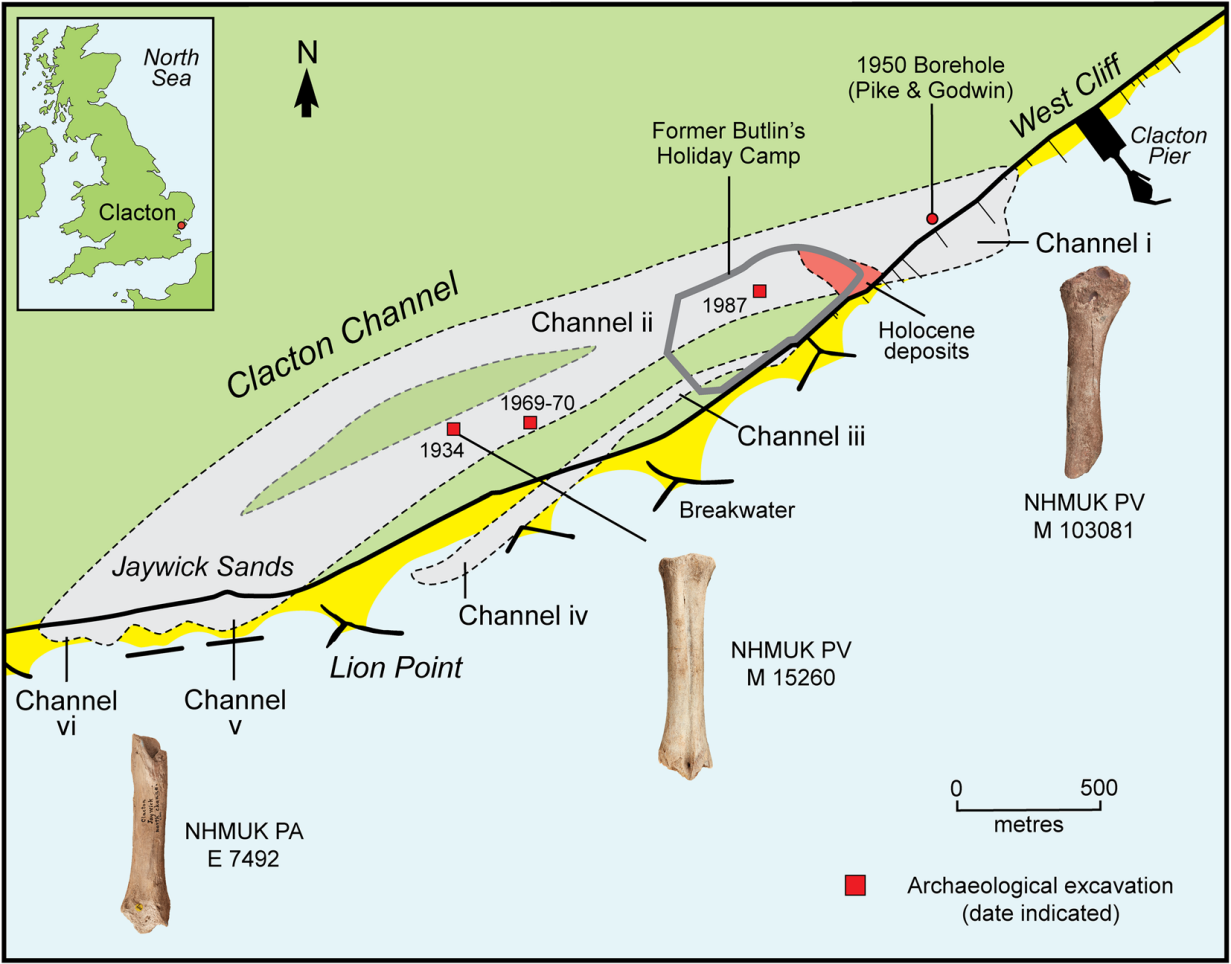
Map of the Pleistocene channels in the Clacton-Jaywick area, showing the findspots of the bone tools (adapted from Allen et al. [ref.^43^, Fig. 12]).

The Thames-Medway channel at Clacton can be traced for 3 km between foreshore exposures at Lion Point, Jaywick, in the southwest and Clacton Pier in the northeast (Fig. 1)^36–43^. The deposits have been investigated in archaeological excavations at Jaywick Sands in 1934^29^, Clacton golf course in 1969–1970^39^ and in 1987 at the former Butlin’s holiday camp at Clacton^40^. Warren identified three parallel channels, two of which (ii–vi, iii–iv) are younger than the channel (i–v) containing the Clactonian artefacts and the richest assemblages of Pleistocene vertebrates^43,44^. At several locations, the Pleistocene sediments are overlain by Holocene estuarine and freshwater deposits^40,43^.

The Clacton Channel is cut into London Clay and has a width of about 300 m and a maximum depth of 17 m. A basal fill of freshwater sands, gravels and marls (~ 11 m) is correlated with the ‘Lower Gravel’ and ‘Lower Loam’ containing Clactonian artefacts upstream at Swanscombe^45^ and the Ebbsfleet Elephant Site^34,35^. Palaeobotanical evidence links the Clactonian to the early temperate zone of the Hoxnian (Ho II), which is characterized by regional mixed-oak forests^46^. Towards the top of the freshwater sequence there is a shift to conifer-dominated woodland marking the start of the ensuing late temperate subzone (Ho IIIa). The freshwater deposits are overlain by up to 5 m of estuarine laminated clay that marks a major marine transgression during Ho IIIb^36,46^.

A rich lithic assemblage from the channel includes cores worked with hard hammers and a range of cutting and scraping tools manufactured from flakes. Much of this material is in primary context with refitting flakes from the golf course site^47^ indicating *in-situ* burial of activity areas along the banks of the river. Associated vertebrate and molluscan remains suggest that human activity took place in a landscape supporting a mix of deciduous woodland and areas of herbaceous vegetation on the floodplain.

The most detailed survey of the Clacton mammal fauna was undertaken by Schreve^48^, who identified 19 mammalian taxa and catalogued 952 specimens dominated by deer (47%), aurochs/bison (29%) and straight-tusked elephant (10%). Horse, lion, wild boar, and two species of rhinoceros, each account for < 5% of the sample.

The bones were collected from locations on the foreshore^36^, as well as from construction sites and archaeological excavations^29,36,39^. A taphonomic study of the faunal remains from Leakey’s excavation^29^ found butchery marks on about a third of the bones (unpublished data) and identified one of the bone tools described in this paper.

## Sample and methods

We studied three putative bone tools from Clacton stored at the Natural History Museum, London, UK (NHMUK). The bison metatarsal (NHMUK PV M 15260) from Trench CII B of Mary Leakey’s 1934 excavation at Jaywick Sands^29^ was recovered from a layer of fluviatile sand and loam (L2) containing abundant Clactonian flint artefacts and butchered large mammal bones. This specimen did not draw attention in Oakley and Leakey’s report^29^, but on closer examination we noted an area of atypical battering and flaking at its proximal end. The second specimen, excavated from the foreshore at Jaywick, was described and figured by Warren (p.129; plate 6)^1^ as a ‘bar-hammer’ (billet). This is a deer tibia (Warren’s No. 47; NHMUK PA E 7492) with battering damage and flaking of the cortical bone. Warren notes ‘There is another similar (not illustrated), which is only battered and flaked down one side’; this is a rhinoceros radius (NHMUK PV M 10381).

Details of the locations and horizons of the deer tibia and rhinoceros radius were not recorded at the time of their discovery. Assigning these specimens to any one of the beds and channels defined by Warren involved two approaches, the first based on preservation type and the second on analysis of sediment attached to the bones for palynomorphs.

The degree of mineralization, colour, post-depositional taphonomic alterations and adhering sediment were assessed visually for bones from various horizons and/or foreshore localities and finds recovered from trenching at Clacton and Jaywick. Sediment samples removed from the medullary cavity of the red deer tibia and adhering to the surface of the rhinoceros radius were prepared for pollen analysis by standard procedures (Supplementary Information)^49,50^. The resulting pollen spectra were compared with pollen analyses from boreholes that recovered samples from Channel i at West Cliff, Clacton^46,51^ and with samples from shorter sequences in temporary sections recorded at the former Butlin’s holiday camp (Channels i, ii and a Holocene channel fill).

The bones were examined with the methods outlined in Bello et al*.*^52^. Initial observations under a binocular light microscope (BLM) with unidirectional lighting identified surface alterations that were recorded on annotated drawings of each bone. We then selected areas for examination with a Focus Variation Microscope (Alicona G5 + Infinite Focus optical microscope) to construct digital three-dimensional models of the features. Interpretations of the surface alterations are based on comparisons with the modern taphonomic collection at the NHMUK^53^ and with bones used in flintknapping and hammering experiments^23,24,54–57^.

## Stratigraphic provenance of the bone tools

Although the bison metatarsal, the deer tibia and the rhinoceros radius are undoubtedly Pleistocene fossils, the only specimen with precise location information is the bison metatarsal^29^ (Fig. 1). Notes detailing the circumstances of the discovery of the rhinoceros radius have yet to be found, but it is likely that it was recovered from a foreshore exposure of Channel i (Fig. 1). Here, the Lower Freshwater Beds are the source of most of the bones and artefacts recovered by Warren. Bones from this locality typically exhibit a distinctive preservation type (Table 1), which is characterized by dark brown to black colour, varying amounts of post-depositional pitting, heavy mineralization and concretions of shelly sand and gravel. In this regard, the rhinoceros radius closely matches other large mammal remains known to have been recovered from the freshwater deposits within Channel i. Freshwater shells in the concreted sediment attached to the radius further establish this link. Moreover, the combination of the dark brown/black colour of the bone, the iron- and manganese-rich sediments adhering to the specimen, as well as the characteristic post-depositional pitting are typical of bones from the Freshwater Beds of Channel i.

**Table 1:** Typical preservation characteristics of bones from different localities associated with Pleistocene channel deposits at Clacton and Jaywick Sands.

Locality	Bone colour, taphonomic alterations and concretion/sediment
Channel i, West Cliff, Clacton (Collected by Brown^58^ and Warren from the cliffs and foreshore exposures of the Freshwater Beds)	Dark brown–black, often heavily mineralized with black sandy concretions containing occasional shell fragments. Common taphonomic alterations include trampling striae, often extensive post-depositional pitting, rounding and abrasion of cortex and break surfaces from fluvial transport
Butlin’s holiday camp (Collected by Warren from builder’s trenches)	Light brown–grey. Superficial post-depositional pitting and trampling striae present on some specimens
Mary Leakey’s trenches at Jaywick^29^	Light grey with varying amounts of orangey-brown iron staining. Cortical surfaces generally well-preserved with a few specimens exhibiting minor post-depositional pitting
Channel iii–iv, Jaywick (Foreshore exposures of Last Interglacial (MIS 5e) channel-fill)	Bones with characteristic white colour in mottled orange-grey silty clay with marine molluscs and reworked freshwater ostracods and molluscs probably derived from the nearby Hoxnian Clacton Channel deposits^43^
Lion Point, Jaywick (Collected by Warren from foreshore exposures)	Light brown–fawn–grey with orangey-brown iron staining. Occasional trampling striae and post-depositional pitting, including etching from plant roots. No concreted sediment

**Figure 2:**
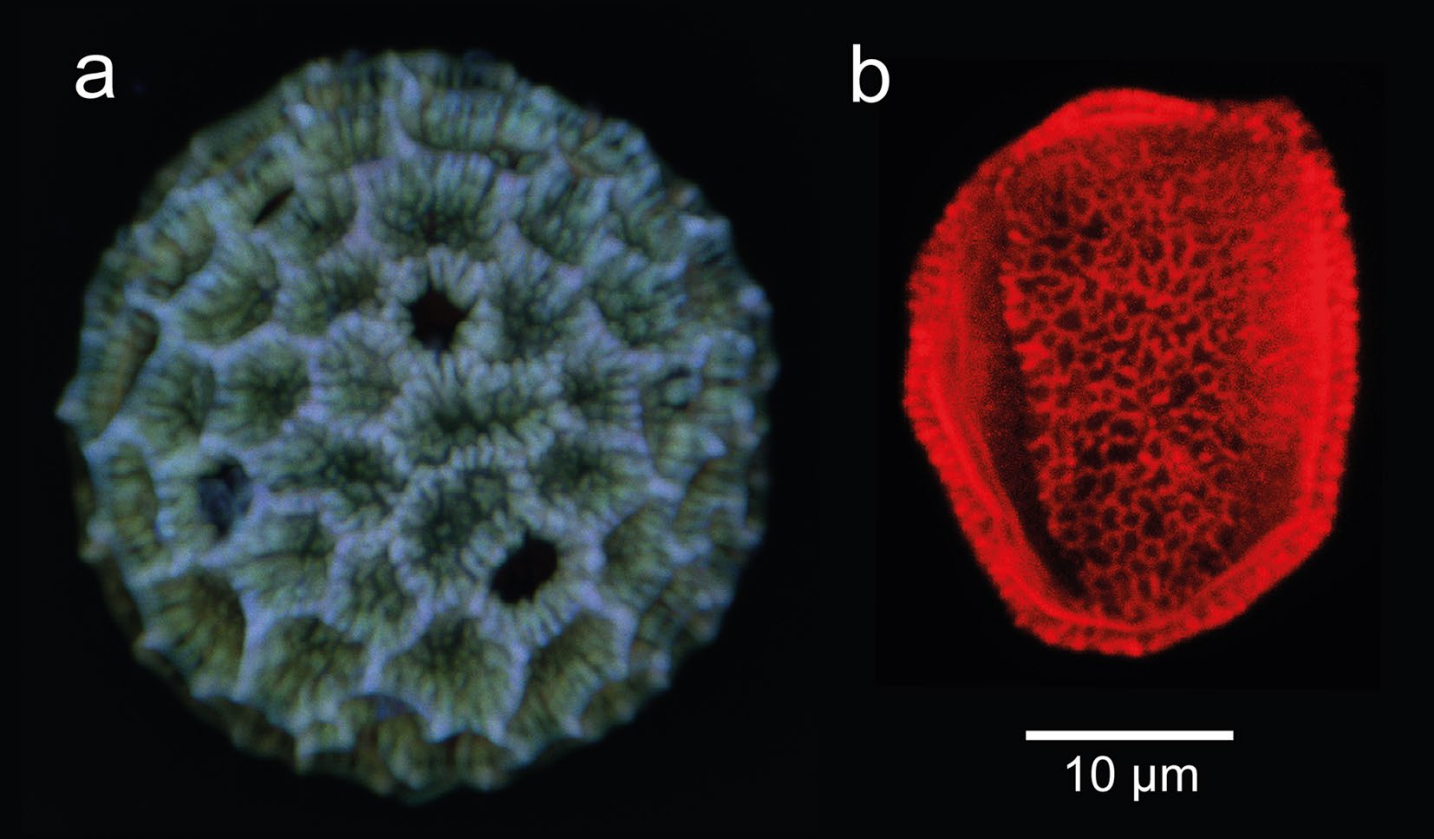
Confocal laser microscope images of pollen grains from sediment adhering to the Clacton bone tools. (**a**) *Persicaria maculosa*, (**b**) Type X. The colour in both images is an artefact of the imaging process. See Supplementary Information for pollen analysis details.

To help determine the provenance of the bones, we decided on a thorough pollen analysis of the sediments attached to them. This is a new approach which provided significant results. The pollen spectrum from the radius (Supplementary Information, Table S1; Fig. 2) provides a tentative vegetational interpretation of a river valley, possibly with backwaters, and nearby grassland and mixed deciduous forest. The spectrum shows clear affinities with British Hoxnian interglacial pollen assemblages, particularly with spectra from the Channel i Borehole B^46^ at Clacton and more importantly with subzone Ho IIb of the Hoxnian parastratotype at Marks Tey^59^.

The red deer tibia was excavated from deposits on the foreshore at Jaywick. The stratigraphy of this area is more complex with at least three distinct channels recorded by Warren: the main Hoxnian channel (v); Channel iv to the east, which is now known to be Last Interglacial in age^43,44^; and to the west there is another post-Hoxnian channel (‘vi’). The post-Hoxnian channels contain estuarine deposits, but they incorporate earlier stone tools and bones reworked from the Hoxnian Channel. A further confounding factor is the presence of Holocene estuarine, alluvial and terrestrial deposits that mantle the Pleistocene sediments and extend onto the foreshore at Jaywick. These deposits also contain reworked Pleistocene material. We consider the tibia to have been reworked from Hoxnian sediments, a conclusion which is supported by the two phases of weathering and abrasion (see below). These features, and the information provided by the pollen analysis (Supplementary Information), are consistent with the bone having been reworked and redeposited into Holocene sediments.

## Description of the bone tools

### Bison metatarsal, NHMUK PV M 15260

**Figure 3:**
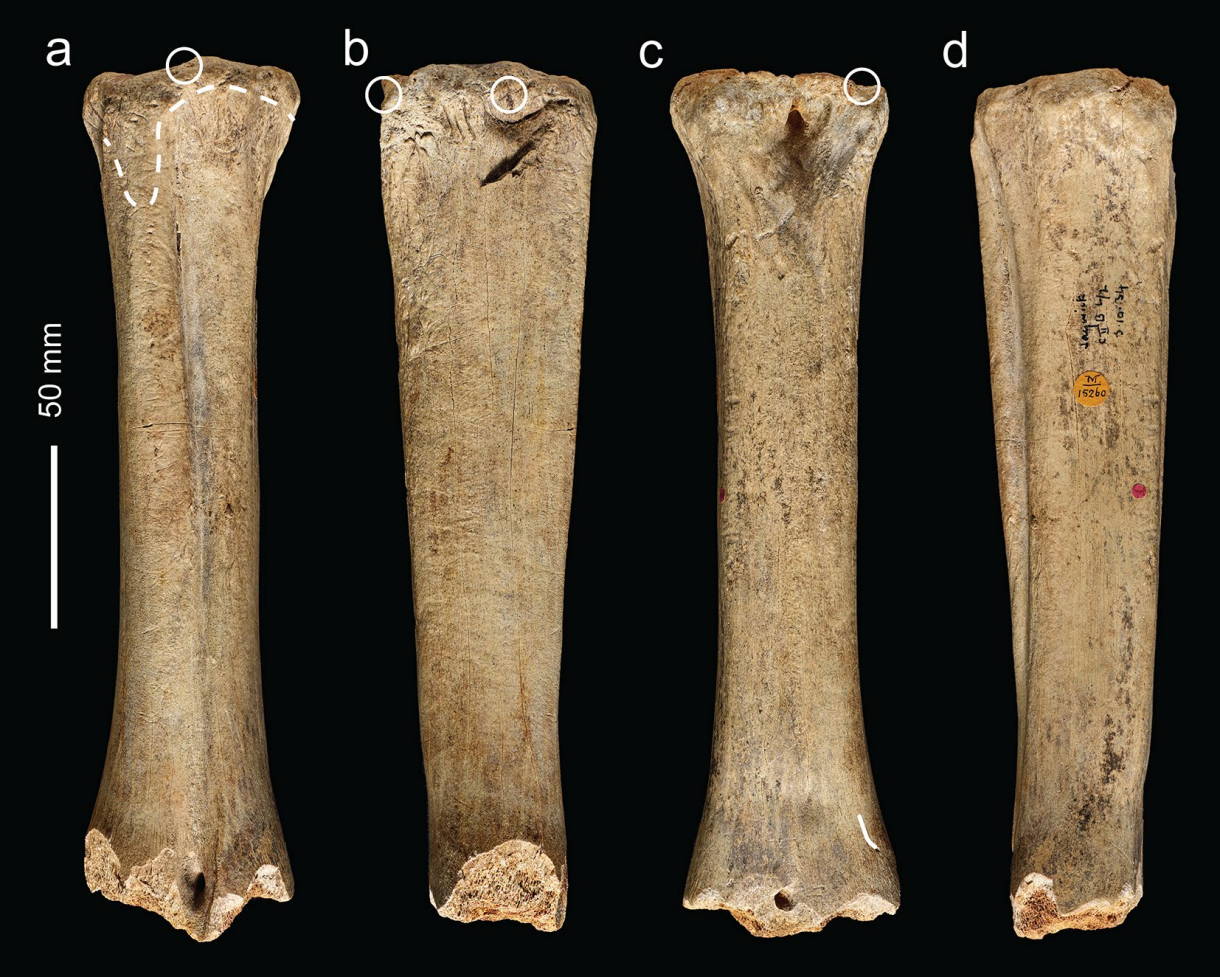
Bovid metatarsal (NHMUK PV M 15260) used as a knapping hammer in dorsal (**a**), medial (**b**), plantar (**c**) and lateral (**d**) view. The area of battering on the dorsal face is bounded by the dashed line, and the circles indicate the points of impact that detached flakes of bone from the proximal end. The cut-mark is indicated by the white line.

NHMUK PV M 15260 (Fig. 3) is an almost complete left metatarsal of a bison (*Bison priscus*) with battering encircling its proximal end. This is a robust bone 240 mm in length from the proximal end to the distal break surface and 61 mm across the proximal end. Except for light lustrous polish, the bone is well preserved. The polish and superficial rounding of the edges of the distal break surfaces appear to be the result of natural processes, probably from suspended particles washing across the bone when it was exposed to flowing water in the river.

Distally, the shaft is truncated by an irregular transverse break surface just below the level of the distal foramen. Modern (excavation) damage includes superficial scratches and a chipped area that has removed a flake of bone from the dorso-lateral part of the distal end. The other parts of this break surface are clearly ancient features as indicated by a slight polishing of the edges and staining that distinguishes it from the modern break. The ancient breaks consist of at least four scallop-shaped flakes that have been removed by pressure emanating from the medullary surface. The flakes expand proximally across the outer cortical surface. No features associated with percussion were observed on this part of the shaft and exactly how this bone became broken is not known, although human activity cannot be ruled out. A cut-mark was noted on the plantar surface near the distal end (Fig. 3c).

**Figure 4:**
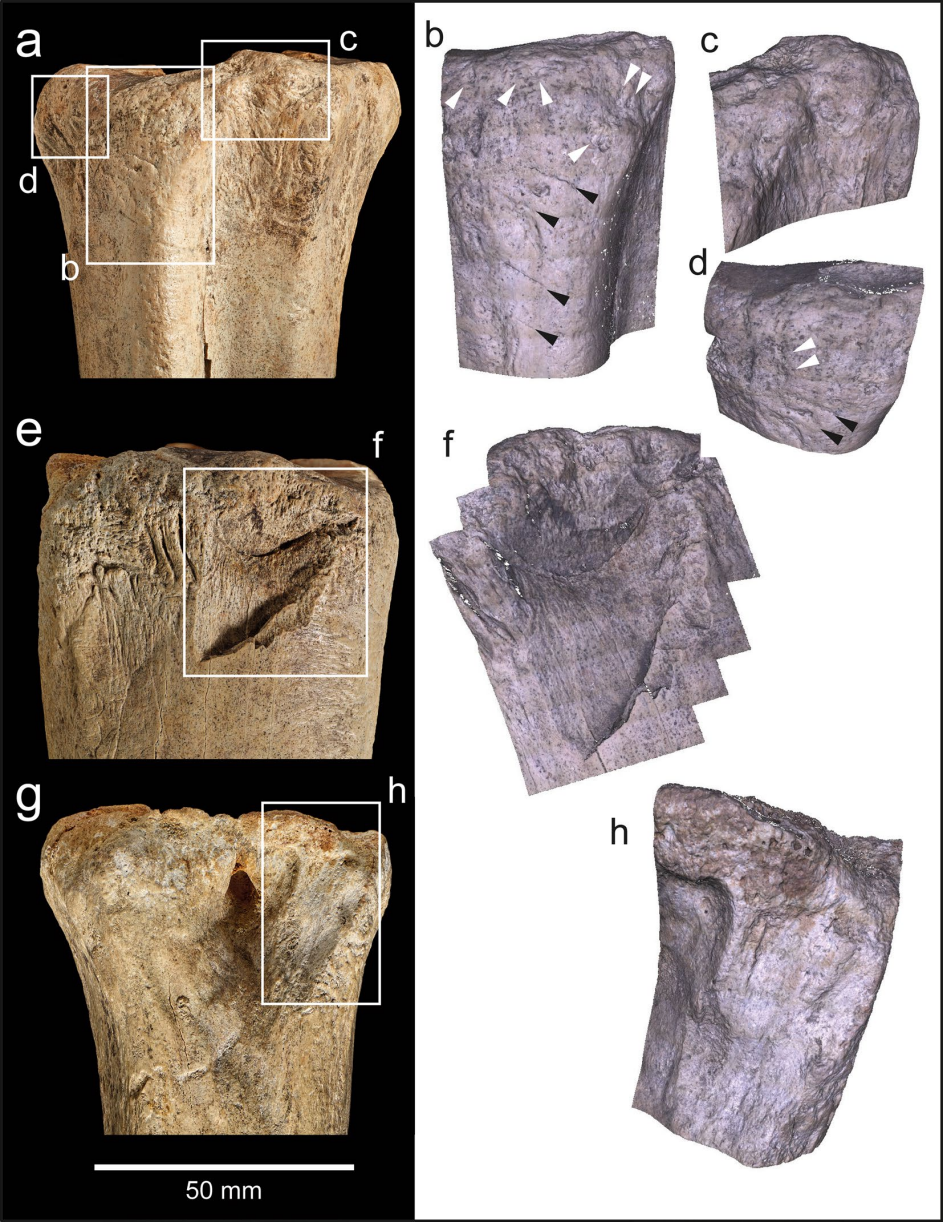
Bison metatarsal NHMUK PV M 15260. Photographs and Alicona images of the battering and flaking of the proximal end of the metatarsal (note rounding along the edge of the articular surface). (**a**–**d**) battering damage on the dorsal surface (black arrows indicate knapping scores; white arrows point to knapping pits), (**e**–**h**) flaking on the medial and plantar surface.

The articular margin of the proximal end is rounded, intensively battered and flaked around most of its circumference (Fig. 4a–h). This damage extends across the adjacent part of the shaft, and distally along the prominent ridge on the mesial side of the dorsal surface. Examination with the BLM reveals that the abraded area is marked by numerous intercutting angular pits and parallel linear grooves (Fig. 4a–d). Observed at low magnifications, the larger grooves on the dorsal ridge resemble chop marks with a slanting cross-section. These features are characteristic of the type of pits and grooves made when a bone is used as a soft hammer to remove flakes from a core or flake, or to retouch a tool edge^57^. Most of the blows were relatively light, although blows that detached the flakes must have been undertaken with considerable force. These more forceful blows detached flakes from the sides of the shaft and from the proximal articular surface. Two large flakes were detached by more powerful impacts directed at the medial face (Figs. 3b,c, 4e–h) with their points of impact located close to the proximal end of the shaft. Flaking of the proximal articular surface includes one blow directed at the angle between the plantar and medial side of the proximal margin, which removed more than half of the proximal articular surface. The other flake scar on the proximal articular surface is smaller (9.5 mm wide) and was removed by a less powerful impact directed against the dorsal edge. On the shaft, a line of at least three impact pits (maximum diameter = 6.5 mm) runs along the ridge on the lateral side of the plantar surface; the distal-most of these pits contains two transverse grooves that are visible under the BLM.

The area of intense battering closer to the proximal end includes numerous small pits as well as scores with an oblique orientation (Fig. 4b). Under the microscope, these marks appear as angular linear gouges and irregular pits with internal striations along the sides of the pits and scores. The morphology of these pits and gouges matches the characteristic features of battering damage inflicted on bone and antler used experimentally when knapping flint^54–57^.

### Rhinoceros radius, NHMUK PV M 103081

**Figure 5:**
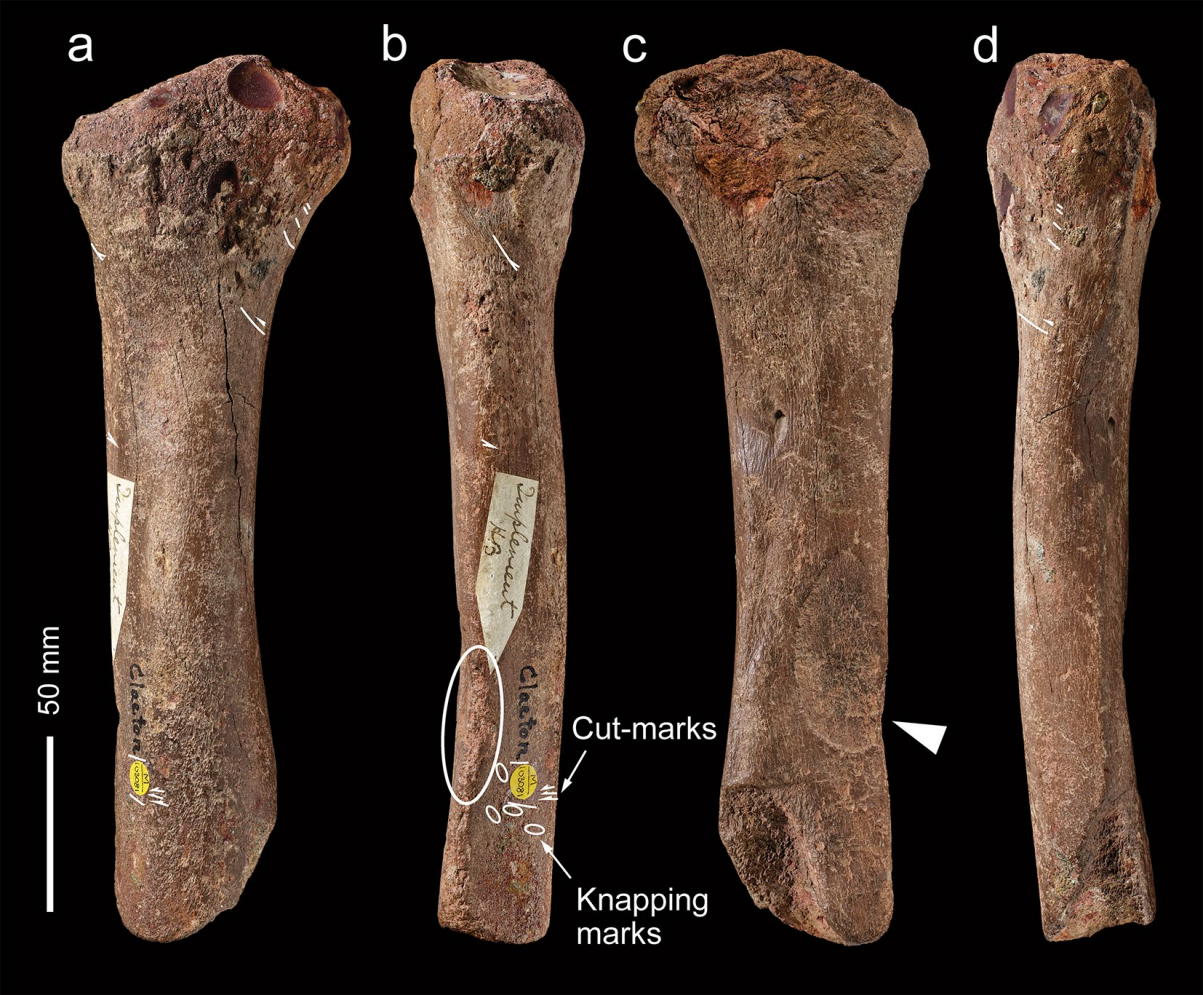
Rhinoceros radius (NHMUK PV M 103081) in anterior (**a**), medial (**b**), posterior (**c**) and lateral (**d**) view. The arrow indicates the point of impact that removed one of the cortical flakes, the large oval encloses the area of battering damage, and small ovals enclose isolated knapping scores. The label (‘*Implement HB*’) points to the damage recognized by Henri Breuil (HB). Burial in an aquatic environment is indicated by impressions of bivalves in the concreted sediment adhering to the proximal end.

NHMUK PV M 103081 consists of the proximal end and most of the shaft of a left radius of a rhinoceros (*Stephanorhinus* sp. Fig. 5). The proximal end is encased in concreted shelly-sand and iron, and manganese oxides have impregnated the bone resulting in its mineralised condition and dark brown colour. Distally, the shaft is truncated by an oblique spiral break that occurred when the bone was fresh; there is no indication as to how the break was initiated. The edges of the break are rounded and shiny, probably from exposure to flowing water.

Most of the bone surface is well preserved, but pits and channels from diagenetic chemical processes and etching from plant roots has removed areas of the surface. An earlier phase of trampling on a gravelly substrate can be identified from random shallow striae on the fresher portions of the shaft.

**Figure 6:**
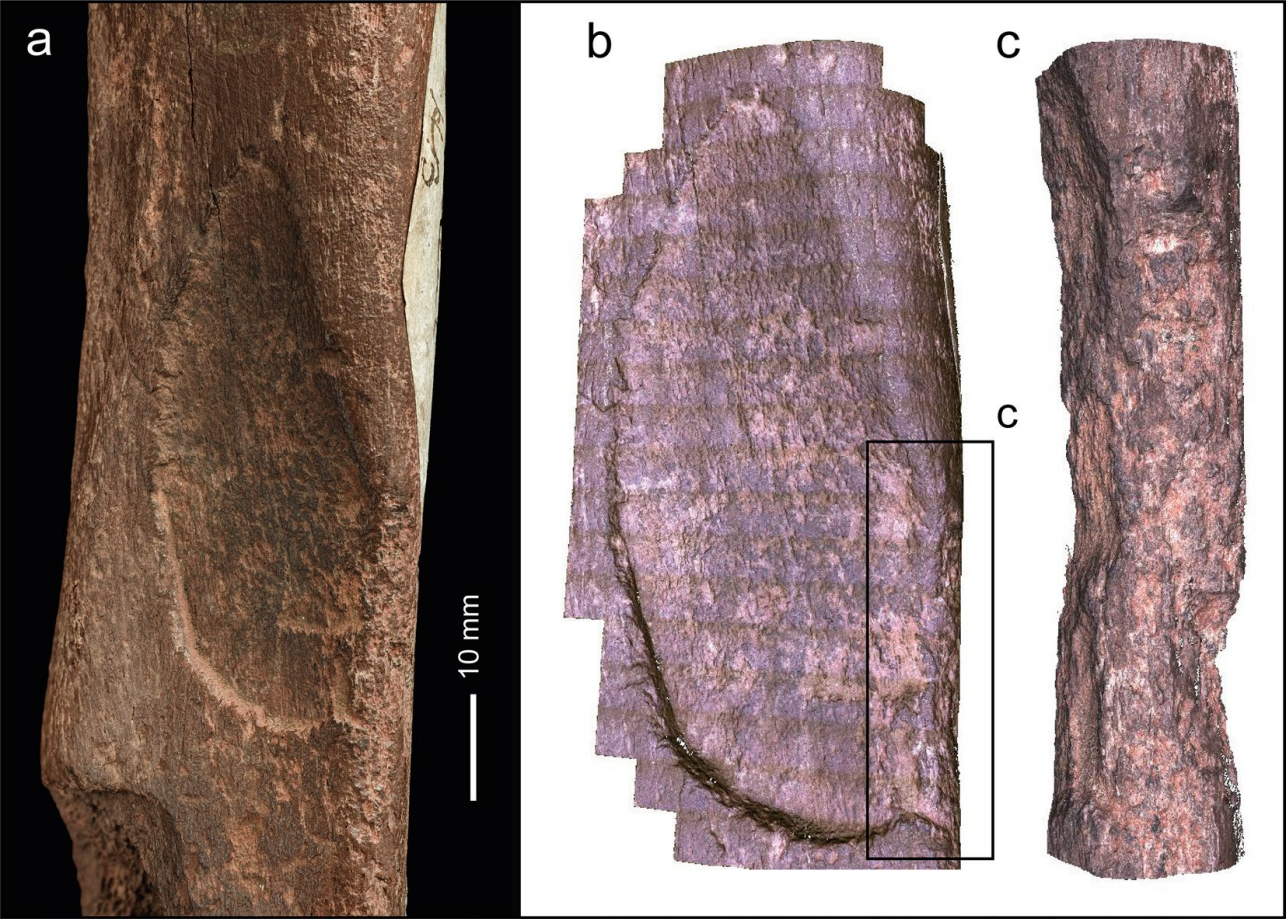
NHMUK PV M 103081—Photograph (**a**) and Alicona images (**b**,**c**) of flake scar and associated battering in a depression created by attrition near the distal end of the shaft. Note the contrast between the area with extensive pitting in the depression along the outer cortical surface and the surrounding unmodified/corroded surfaces.

Humans have modified the bones in two ways. The first is from butchery that resulted in filleting cut-marks at several locations on the shaft (Fig. 5c). The second is a hollow with battered bone and associated flake scars located towards the distal end of the shaft at the angle delimiting the posterior and medial faces of the shaft (Fig. 5b,c). The area of attrition is lozenge-shaped and abraded to a depth of ~ 4 mm (Fig. 6). Figure 6c shows a focus variation Alicona microscope image of the battered area. Although the base of the features is obscured by matrix in the crevices, the damage can be seen to consist of intercutting pits and grooves that are consistent with sustained and focussed battering against a hard object.

The abraded zone is also the origin of two flake scars that have removed cortical bone from the posterior and medial surfaces of the shaft. The flake scar on the medial surface is small and triangular in shape, whereas the flake scar on the posterior face is considerably larger, measuring 59 × 27 mm (Fig. 6a,b). Features of the larger flake scar include ripples and a terminating hinge-fracture, which indicate that the flake was removed by percussion (Fig. 6b,c). These impact features exhibit diagnostic characteristics of battering damage inflicted on a fresh bone when it is struck against a sharp stone edge. Varying amounts of force can be inferred, with the chipping and pitting being indicative of relatively light blows, whereas the removal of larger flakes suggests more forceful actions^21,23^.

### Red deer tibia, NHMUK PA E 7492

**Figure 7:**
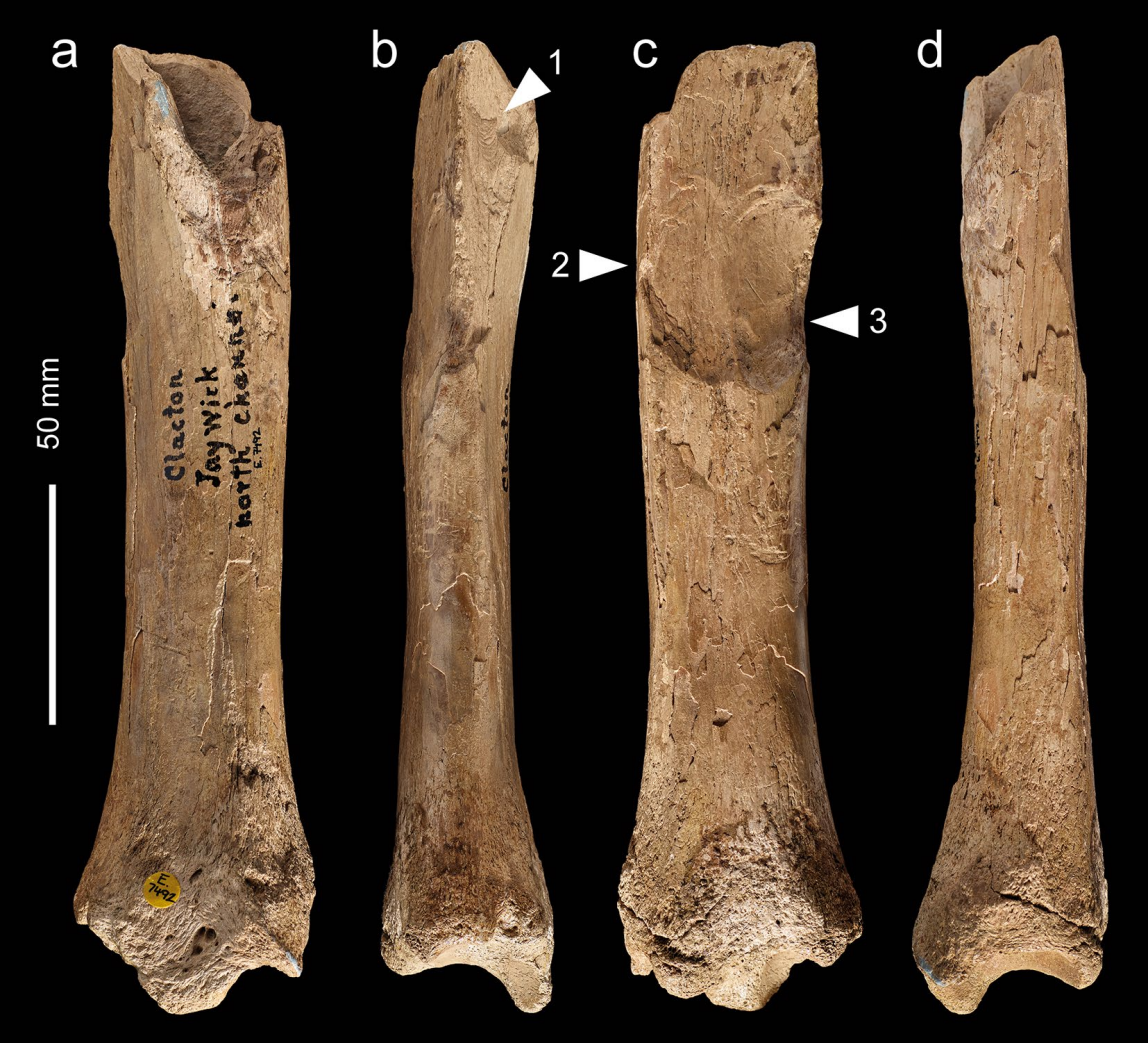
Red deer tibia (NHMUK PA E 7492) in anterior (**a**), lateral (**b**), posterior (**c**) and medial (**d**) view. The arrows indicate the direction and sequence of impacts that removed the cortical flakes.

**Figure 8:**
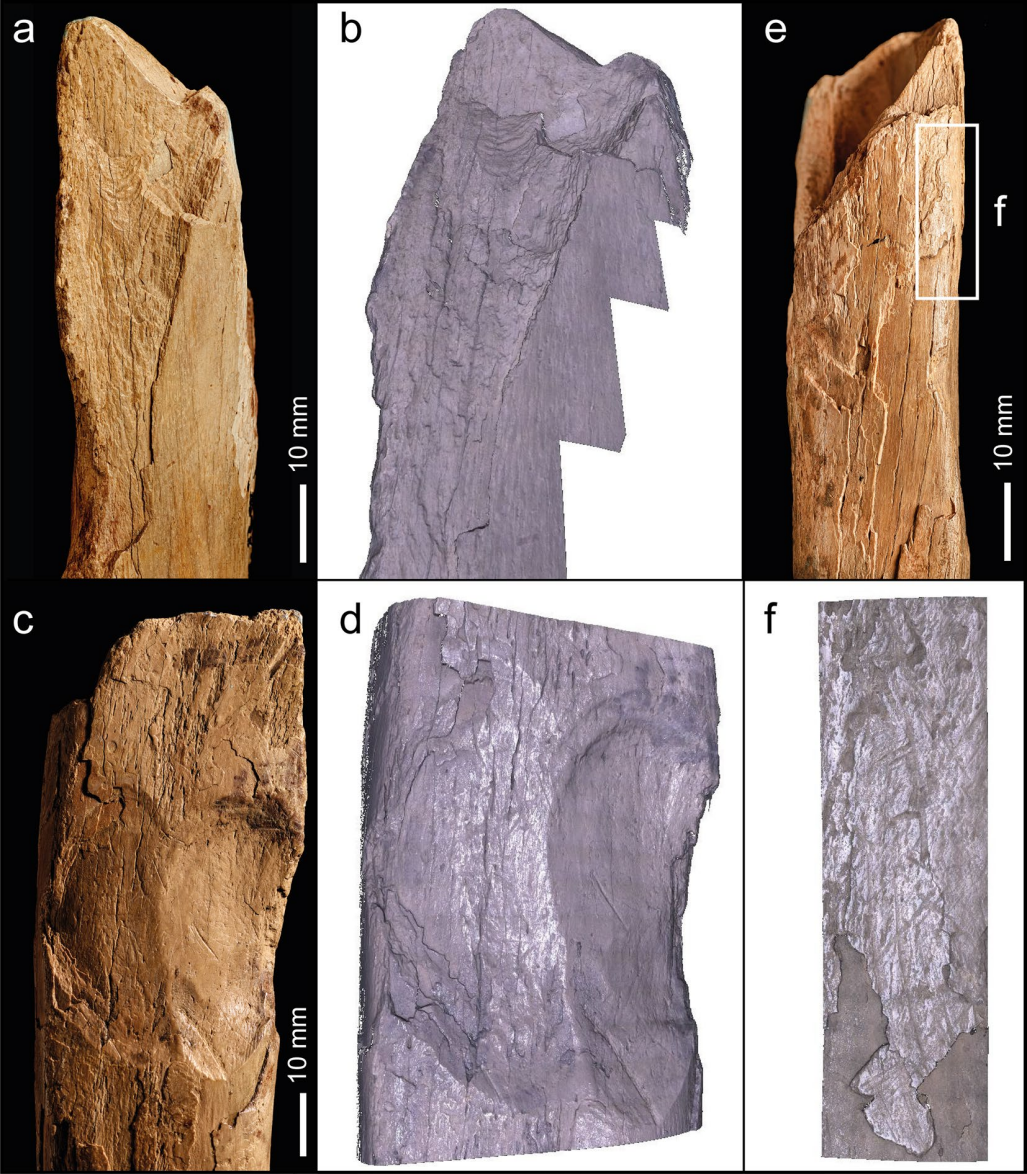
NHMUK PA E 7492—Photographs and Alicona images of flake scars (**a**–**d**) and post-depositional abrasion (scratches and polish), the latter occurring on both flake scars and on weathered surfaces. Details of the weathering show the sequential loss of layers of the outer surface of the cortical bone (**e**,**f**).

NHMUK PA E 7492 is a shaft and distal end of a right tibia of a red deer (*Cervus elaphus*; Fig. 7). The proximal end is fractured with three overlapping flake scars (Figs. 7b,c, Fig. 8a–d) with morphological characteristics (ripples and hinged terminations) consistent with the removal of flakes by percussion. The first flake removal extends from the oblique spiral break surface at the proximal end of the shaft where it is truncated distally by two opposing flake scars (Fig. 7b,c). The second flake scar is the result of a blow directed against the medial side of the shaft. It extends mid-way across the posterior surface where it is truncated by the third flake scar that was initiated by a blow on the opposite side of the shaft.

The size of the scars indicates they were generated when the shaft was struck with considerable force against a hard object. Although it is possible to locate the points of impact, any fine details, such as pitting or similar impact features, have been lost to at least two phases of weathering and spalling of the bone surface indicated by superficial scratches, weathering cracks, rounding and a glossy polish (Fig. 8e,f). The rounding and polished areas are particularly noticeable on the edges of breaks and on the ridges within the flake scars.

These alterations appear to have taken place in two phases. The first phase includes weathering and sediment abrasion when the bone was exposed to the elements as well as being transported by flowing water. After a period of burial, the tibia was subsequently re-exposed. During this second phase of exposure and weathering, substantial areas of surface bone flaked away, and the newly exposed areas and break edges were further rounded, polished and marked by random striae from movement and pressure. Finally, the bone was incorporated in a Holocene grey silty-clay, which filled the medullary cavity.

## Discussion

The pattern of damage allows the specimens to be divided into two groups:

1. The bison metatarsal with flaking and attritional rounding of its proximal end.
2. The rhinoceros radius and deer tibia with flakes detached from the outer cortical surface of the shaft.

The pitting and scoring on the bison metatarsal and the large flake scars on the rhinoceros radius and deer tibia cannot be explained as resulting from natural taphonomic processes, such as trampling, weathering or carnivore action. The modifications can be interpreted with reference to experiments and comparisons with bones from Palaeolithic assemblages that have been used in heavy-duty knapping and other percussive tasks.

### Experimental comparisons

**Figure 9:**
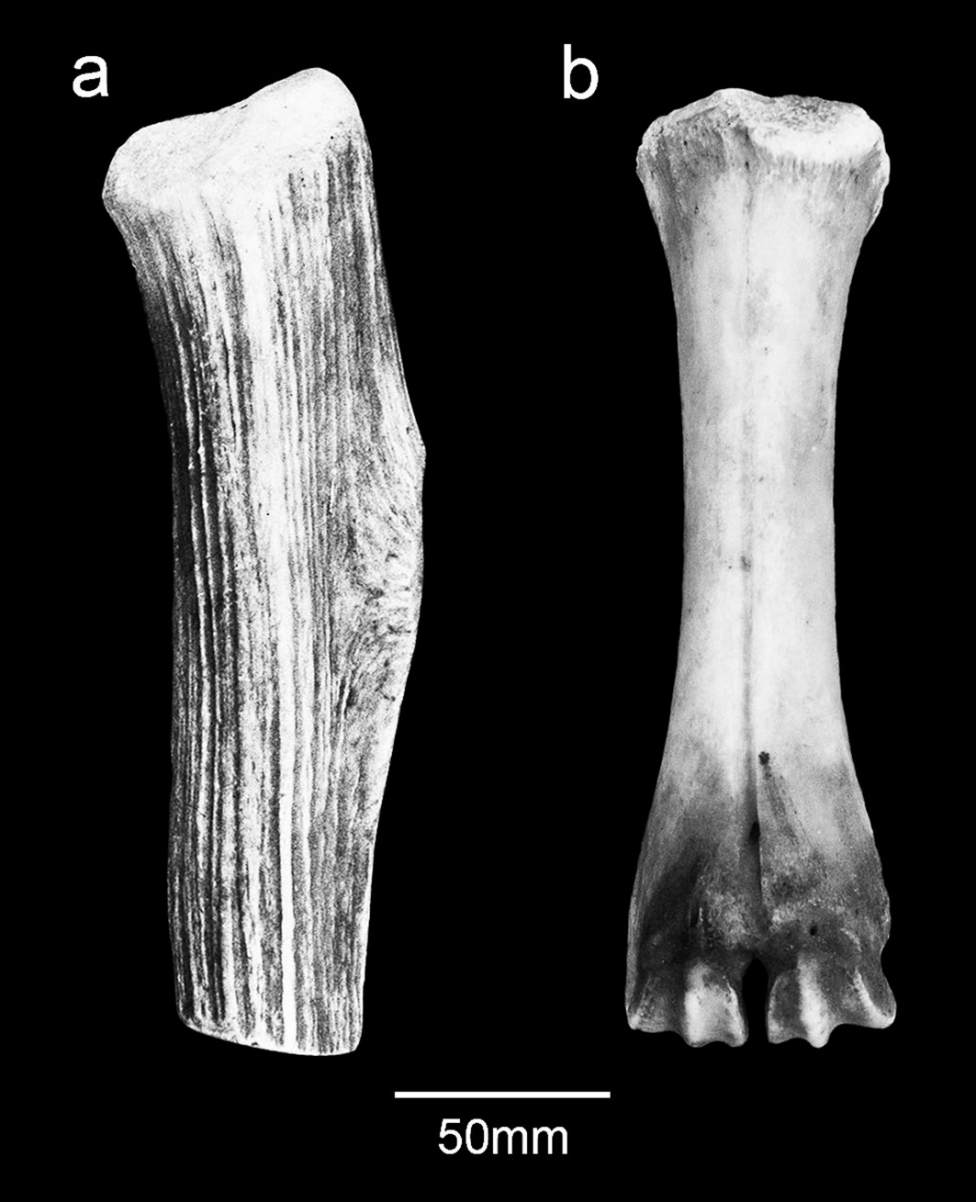
Antler (**a**) and cattle metacarpal (**b**) used as hammers in experiments to knap handaxes. Note the similarity in the distribution of attrition and rounding of the proximal end of the cattle metacarpal with that of the bison metatarsal from Clacton (Fig. 3a).

The modifications on the bison metatarsal can be interpreted from an experimental flintknapping programme undertaken by Francis Wenban-Smith and John McNabb at Boxgrove (UK)^26,56^. The experiments replicated the finely shaped early Middle Pleistocene Boxgrove handaxes using different types of ‘hard’ (stone) and soft organic (antler or bone) percussors (Fig. 9). The aim of the experiments was to establish metrical attributes of the resulting flakes that could be used to identify the percussor types employed to knap the handaxes at the site. The knapping process was divided into two stages, ‘roughing-out’ and ‘finishing’, with the knapper switching from hard hammers (quartzite pebbles, flint beach pebbles and cortical flint pebbles) during the roughing-out stage to soft hammers (trimmed antlers shaped like batons and cattle metapodials) at the shaping stage. The results of the experiments were successfully applied to the Boxgrove archaeological assemblage, which identified a clear separation in the metrical attributes of the waste flakes detached with soft hammers from those knapped with hammerstones. The use of soft hammers at Boxgrove was subsequently confirmed by the identification of numerous bone percussors and at least three antler soft hammers from different locations and horizons, suggesting that organic soft hammers were an important curated component of Acheulean tool kits as much as half a million years ago^27,60^.

The experiments directly relevant to the interpretation of the bison metatarsal are those undertaken with cattle metapodials. Cattle metapodials were selected as the most suitable bones for soft hammers because they are naturally shaped like a baton, and the bone itself is typically dense with thick cortical bone. In use, the metapodials were held towards the distal end of the shaft and the proximal end was struck with varying degrees of force directed against the edge of the handaxe roughout to remove thinning flakes. Not only did these experiments demonstrate that bovid metapodials make effective flintknapping tools, but they also allowed examination of damage to the knapping area, which consisted of attritional rounding of the proximal end from inter-cutting gouges and angular pits (Parfitt, pers. obs.).

Microstriations were also observed marking the walls of the pits and grooves, and other areas with microstriations extended across the surface of the bone beyond the indented features. Another characteristic of these knapping tools is the occasional presence of small slivers of lithic debris embedded in the surface of the knapping area. Although no flint debris was identified in the Clacton specimen, the overall pattern of damage observed in the experimental knapping hammers is otherwise identical to that of the Clacton bison metapodial (compare Fig. 9b with Fig. 3a).

The damage on the rhinoceros radius and deer tibia from Clacton is less easy to interpret. In both cases large flakes have been detached from the outer cortical surface of the shafts. We investigated five hypotheses to account for the flaking on these bones: (1) they were struck with the intention of breaking the bones to access the marrow; (2) they were knapped to produce usable bone flakes; (3) they were struck against wedges, or other intermediate tools; (4) they were used as hammers to break open marrow bones or (5) as soft percussors to knap flint.

The first hypothesis can be rejected because the percussion features did not result in clean breaks that opened the marrow cavity. The most efficient technique to extract marrow relies on simply striking the bone with a hammer (usually a stone) when the bone is held in the hand or placed on an anvil, or by striking the bone against a stationary anvil^61–63^. The precise methods used to break marrow bones vary between different cultural groups^64^, but the blows typically result in a circular or oval impact area with flakes that are driven into the medullary cavity at the point of impact^65–67^. Successfully breaking open the marrow bone can usually be achieved by a single, carefully judged blow^61,62^.

Deliberate knapping of the bones to detach sharp, usable flakes (hypothesis 2) is also unlikely. This is because the damage is not consistent with controlled knapping of bones^9,10^, as shown most clearly in the rhinoceros radius where the flaking originates from an area of intense attrition resulting from repeated blows having removed tiny chips of bone from the impacted area. It is also significant that the size of many of the flake scars suggests that the detached flakes would have been too small to have functioned as cutting or scraping tools. We also examined the Clacton faunal collection for evidence that larger bones of rhinoceros and elephant had been knapped to make tools or to remove usable bone flakes, but no examples were found. This suggests that intentional knapping of bones to make tools was not undertaken at Clacton.

If the three Clacton bones had been used as hammers to strike wedges, adzes, or other intermediate stone tools (hypothesis 3), the associated flintwork assemblage would be expected to include such flint tool types. Although wedges and adzes have been identified by Keeley^47^ as an element in the Acheulean tool kit at Hoxne, both Keeley and McNabb’s^3^ analyses of the more extensive Clacton flint assemblage failed to identify similar pieces. This suggests that wedging and adzing tasks were not undertaken at Clacton.

An alternative explanation for the flaking on the Clacton bones is that the damage is incidental to their use as hammers, which were used either to break open bones for marrow (hypothesis 4) or as soft hammers to knap flint (hypothesis 5). To distinguish between these two possibilities, we compared the damage on the Clacton specimens with features on long bones used in experiments to break marrow bones and knap flint^23,24^, and with Palaeolithic knapping hammers where the long-bone shaft had flaked and shattered during use.

The first set of experiments were undertaken to throw light on the enigmatic ~ 300,000-year-old bone hammers from Schöningen, Germany^15,17,18,22,24^. An intriguing component of the bone tools from Schöningen is an assemblage of horse metapodials with flaked and rounded epiphyses from their use in heavy percussive tasks. Voormolen^15^ attributed the damage to their use as hammers in ‘heavy duty’ flint-knapping tasks. More recently van Kolfschoten et al*.*^17^ noted an absence of flint chips embedded in the working area of the epiphyses and suggested that these metapodials had been used as hammers to crack open marrow bones.

Recent experiments^23,24^ support this suggestion by showing that horse metapodials can be effectively used as hammers to knap flint and as percussors to break marrow bones, with distinctive damage patterns associated with the two tasks. The metapodial epiphyses used to crack marrow bones exhibit polishing and rounding from compression of the bone matrix. These features are accompanied by chipping and indentations created when the hammer struck a sharp bone edge or an angular protuberance on the surface of the marrow bone. Larger flakes were also detached from the epiphyses of some of the metapodials. A different type of damage was seen in the bones used to knap flints, which are marked by attrition from intercutting angular pits and scores. The distribution and microscopic features of use-wear on the bison metatarsal from Clacton are identical to those of the horse metapodials used experimentally to knap flint.

### Archaeological comparisons

The flaking on the Clacton radius and tibia are atypical of the type of damage normally associated with bones used as knapping hammers and as retouchers. The ‘search image’ for these tools is provided by classic Mousterian examples first described by Martin^68,69^ and found subsequently at numerous Middle Palaeolithic sites^22,54^. These knapping tools are commonly made on thick cortical bone, typically long bone shaft fragments, which were used in light duty tasks to shape and re-sharpen relatively small flake tools. The resulting damage consists of localized areas of shallow pitting and attrition. However, another category of knapping tool is more commonly found at sites where larger flakes were detached from cores and handaxes, and in assemblages where knapping intractable lithic raw materials required a greater amount of force. This type of ‘non-classic’ retoucher includes examples from sites such as Boxgrove^60^ and Payre in France^21^.

**Figure 10:**
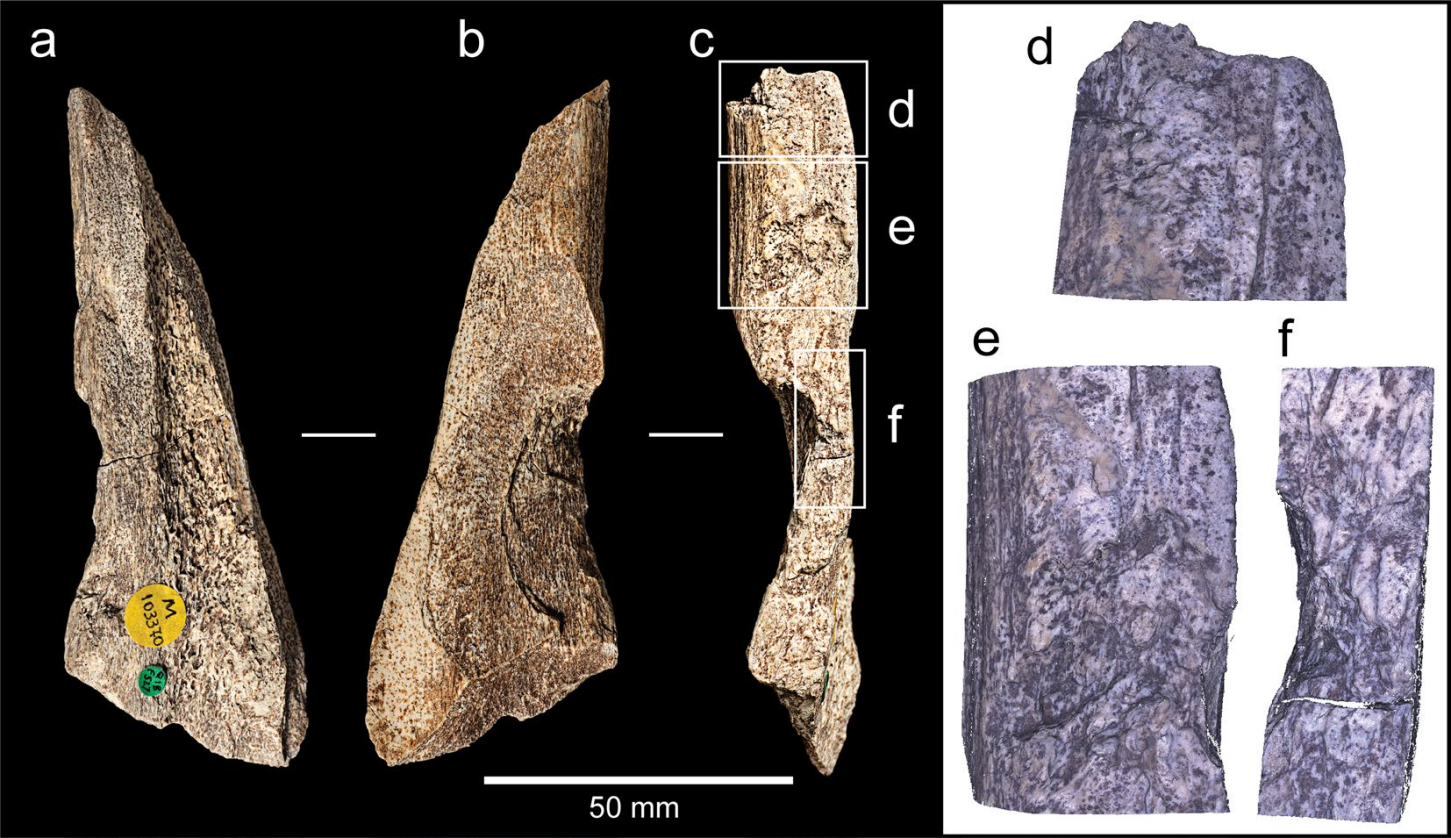
Photographs and Alicona images of a long-bone shaft fragment (NHMUK PV M 103370) from Boxgrove (site Q1/B, find number F527) exhibiting large flake scars associated with knapping damage on the outer cortical surface (**d**–**f**).

An example from the Boxgrove Waterhole Site (Q1/B)^70^ shows the key features of this type as represented by a shattered long-bone shaft fragment (NHMUK PV M 103370) with large flake removals associated with knapping damage (Fig. 10). The fragment is 123 mm long and comes from the middle of a shaft with thick (9 mm) cortical bone, probably from a large bovid, equid or rhinoceros. The fragment has two faces at right angles to each other with the knapping areas aligned along this angle. The knapping pits are grouped in at least four areas (Fig. 10). Blows at one of these areas removed large flakes from the cortex, the largest of which is > 66 mm wide, which was followed by a second flake removal leaving a scar measuring ~ 38 mm wide. The bone shattered during use, and this fragment is essentially a large flake resulting from the terminal impact. Several similarly broken knapping percussors occur in the Boxgrove assemblage^60^. The interpretation of the Boxgrove bone and antler hammers is that powerful striking actions were sometimes required to remove large flakes from handaxe roughouts^27,56,60^. These actions resulted in deeper pits and scores than those seen in most Middle Palaeolithic retouchers, and in some cases the knapping tools flaked or broke during use^71^.

**Figure 11:**
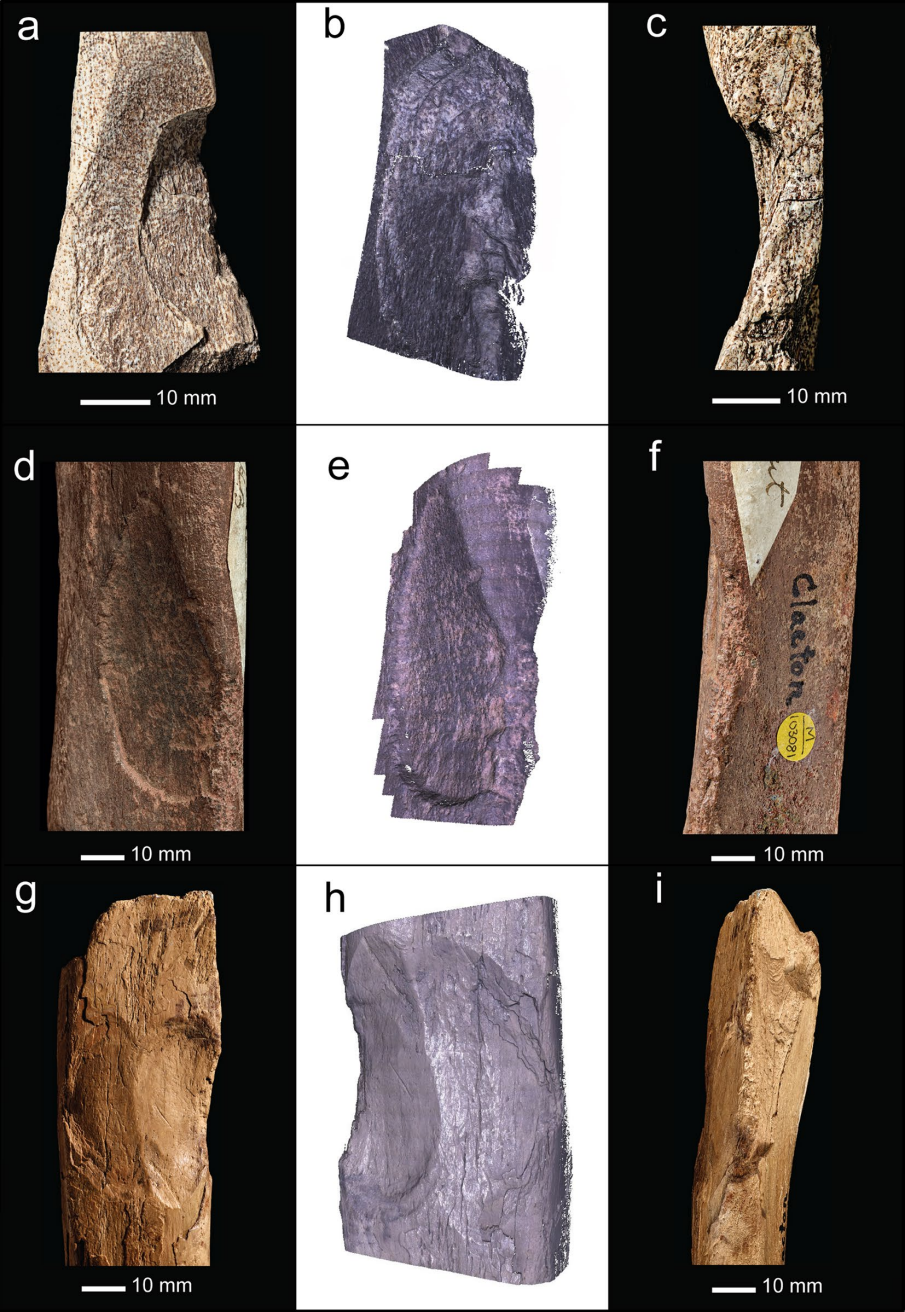
Comparison of flaking of the Boxgrove percussor fragment (**a**–**c**, NHMUK PV M 103370) with flake removals on the shafts of the rhinoceros radius (**d**–**f**, NHMUK PV M 103081), and deer tibia (**g**–**i**, NHMUK PA E 7492) from Clacton. The Alicona images are oriented to show battering damage, which is well preserved in the Boxgrove example but partly covered by matrix in the rhinoceros radius; it is not possible to estimate the extent of damage to the tibia because weathering has removed the outer surface of the cortical bone in the critical areas.

A comparison of the flake scars on the Boxgrove shaft fragment with those of the Clacton tibia and radius (Fig. 11) illustrates the close similarity of the flaking on this specimen with that of the flaking on the Clacton shafts, particularly the radius which also preserves pitting on the surface where the flake originated.

Close parallels with other flaked and broken bone knapping hammers can also be found in the assemblage from Payre^21^. At Payre, knapping tools are associated with an early Middle Palaeolithic (MIS 8–7) lithic assemblage that includes heavy duty tools, as well as bifaces and pebble tools made on various intractable rock types. The retouchers include several examples with cortical notches, demonstrating a powerful striking action was used to knap the lithic tools. An illustrated example [ref.^21^, Fig. 8, p. 112] shows a similar flaking pattern to that of the Boxgrove and Clacton specimens. This shaft fragment is from the distal part of a horse metapodial and has been interpreted as a broken retoucher. It exhibits angular scores and pits (the ‘retouching area’) associated with a large flake scar (maximum dimension ~ 35 mm).

### Soft hammers and Clactonian knapping techniques

The Clacton bone hammers were used in two different ways: the first, with the striking area located at the proximal end of the bison metatarsal (this can be matched with the similar use of metapodials in the Boxgrove and Schöningen knapping experiments); the second, with the working area located further down the shaft of the radius and tibia (this use-wear can be matched with archaeological knapping hammers from Boxgrove and Payre). Both the metatarsal and radius exhibit key features that link these bone tools to knapping tasks. The metatarsal is well preserved, and although surface features are degraded on the radius, both exhibit characteristic knapping pits and attrition accompanied by the occasional removal of larger cortical flakes. A similar mode of use is inferred from the flaking on the tibia, although interpretation is hampered in this case by poor preservation of the cortical surface. The selection of long and stout limb bones is also noteworthy, although the significance of the possible intentional removal of either the proximal (tibia) or distal epiphysis (metatarsal, radius) is unclear.

**Figure 12:**
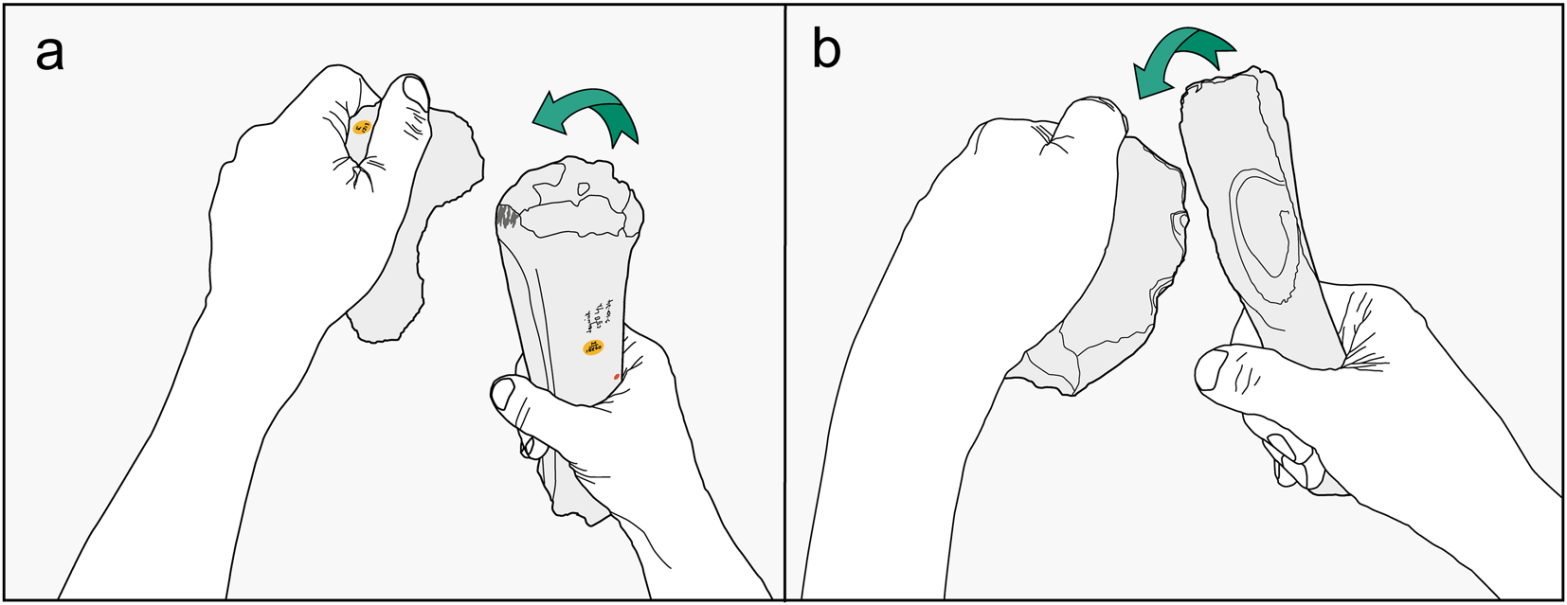
Reconstruction of Clactonian flintknapping using bone hammers to modify flakes struck from a core with a hard hammer. (**a**) bison metatarsal NHMUK PV M 15260, (**b**) deer tibia NHMUK PA E 7492.

The battering marks on the Clacton bone tools provide further clues as to how they were handled and used to knap flints (Fig. 12). Looking at the variation in the size and depth of the battering marks, it can be inferred that most of the blows were light, whereas considerable force would have been required to remove large cortical flakes. The lighter blows may have been aimed at finer-flaking during shaping or resharpening flake-tools, whereas the more powerful blows would have detached larger flint flakes.

Further support for the use of bone hammers at Clacton is provided by the presence in the lithic assemblage of rare flint flakes with flat diffuse bulbs, which appear to be the result of soft hammer percussion^cf.^^72^. An example illustrated by Singer et al*.* [ref.^39^, Fig. 3, 32, nr. 1315] is a ‘side scraper made on a cleanly struck flake with a diffused bulb of percussion and a plain striking platform’, which is a strong contender for a flake produced by soft direct hammer percussion.

The significance of possible soft-hammer flakes in the Clactonian has not been commented on previously, but the apparent rarity of such pieces in the Clacton collection may suggest that organic hammers were used only infrequently and mostly for lighter knapping tasks which produced smaller waste flakes. The scarcity of soft-hammer flakes at Clacton may be due to other factors, such as recovery biases in un-sieved assemblages that result in the under-representation of small flakes produced during shaping/retouching tasks, combined with difficulty of distinguishing these soft hammer chips from those detached with a stone hammer. The Clacton artefacts were also buried in a fluvial situation, where winnowing processes are known to lead to a depletion of hard hammer flakes^73^.

The working areas on the rhinoceros radius and bison metatarsal are both heavily worn; this is consistent with repeated and intensive use (the level of attrition cannot be scored in the deer tibia due to weathering). This pattern is atypical for most Palaeolithic bone retouchers, which appear to be opportunistic (informal or ad hoc) tools that were picked up where they were used and discarded on the spot after a short use-life in retouching a single lithic tool [e.g., ref.^54^, but see ref.^60^]. In contrast, the intensity of the damage on the Clacton metatarsal and radius suggests a longer use-life with the tools having been employed several times in the shaping and/or re-sharpening of more than one flake tool. Whether these bone tools were part of a curated tool kit will be difficult to determine.

## Lower Palaeolithic organic soft hammers

The use of soft hammers has been linked to advanced cognitive abilities of handaxe makers relative to earlier *Homo* and contemporary populations that relied on simpler lithic technologies. The origin(s) and spread of this innovation are currently the subject of active research^22^. The use of soft organic hammers underpins the fundamental division in early stone tool technologies between the simplest Mode 1 technologies (e.g., Oldowan, Clactonian) that employed hard hammers to detach flakes from cores, and later Mode 2 handaxe technologies that utilized more controlled knapping techniques including the use of soft organic percussors during the later stages in its evolution. The Clactonian examples are significant in these debates as they represent the earliest known soft organic knapping hammers from a Mode 1 context.

The challenges of studying early organic knapping tools include problems of distinguishing them from tools used to perform different percussive tasks, such as processing plant foodstuffs. These studies are also complicated by the problem of differentiating naturally-modified bones from those modified by hominins [ref.^74^, pp.338–347]. These problems are illustrated by some of the oldest purported bone tools with percussion damage from Early Pleistocene contexts at Olduvai Gorge (Tanzania). At Olduvai, the earliest bone tools used for percussion tasks are from Bed II. They are associated with a developed Oldowan lithic industry and date from about 1.8 to 1.2 million-years ago. The sample includes several bones with puncture marks that were initially interpreted as anvils on which to pierce skin^75,76^. A subsequent study by Backwell and d’Errico^10^ came to a different conclusion with the suggestion that the punctures on a giraffe astragalus (BKII 2933) and an elephant patella (FLKII 884) resulted from ‘hammering tasks, most likely on intermediate stone wedges used to split bones, fruit or wood’ [ref.^10^, p.110]. The puncture marks also resemble knapping marks on hand-held hammers, but a recent suggestion by Pante et al.^9^ links the triangular pits on the elephant patella to crocodile bite-marks. Pitted bones from later levels at Olduvai include a proboscidean astragalus (JK 3109) from Bed III (~ 1.15–0.93 Ma) that may have been used as an anvil for bipolar percussions, and a hippopotamid humerus from (PDK 895) from Bed IV (~ 0.93–0.8 Ma) has battering damage that may have occurred when the bone was used as a hammer or anvil^9^.

At Konso (Ethiopia) a sequence of sites records advances in knapping technology with roughly-made early Acheulean handaxes occurring in deposits dating to ∼1.6 Ma, and the development from ∼1.4 to 1.25 Ma of a prepared core technology and the early adoption of a specialized technique (the Kombewa method) that resulted in handaxes with thinner tips and increased symmetry and comparatively straight edges^77^. Further advanced techniques appear about 0.85 Ma with more intensive and finer flaking leading to even greater levels of symmetry and a substantial reduction of thickness. It is currently unclear whether soft hammers were used to remove the shallower invasive flakes from the blank surfaces. Intriguingly, the bones from Konso include two pieces (KGA10 ZA62, humerus shaft of a large mammal; KGA10 ZA68 distal humerus of small-sized bovid) with clusters of percussion pits [ref.^78^, Figures 13–14] that closely resemble damage left on bones used as retouchers and hammers to shape and resharpen lithic artefacts.

An alternative view is provided by Clément^79^ who questions whether the handaxes from Isenya (Kenya), dated to ~ 960 ka, had been manufactured with soft organic hammers as suggested by earlier experimental studies. Her experiments show that Isenya handaxes can be replicated with softer stone hammers and by adapting the knapping style to detach elongated thinning flakes.

The evidence for organic knapping tools in the Lower Palaeolithic beyond Africa is also meagre, although evidence from Europe and the Levant suggests that organic knapping tools were employed to manufacture finely-flaked Acheulean handaxes from at least the early Middle Pleistocene (Table 2).

**Table 2:** European Lower and early Middle Palaeolithic sites^17,18,20,21,71,88^ with soft hammers/retouchers associated with different lithic technologies.

Marine isotope stage (ka = thousand years ago)	Core-and-flake industries (Mode 1)	Handaxe industries (Mode 2)	Prepared core industries (Mode 3)
7 (243–191 ka)			Biache-Saint-Vaast
8 (300–243 ka)			Payre (MIS 8–7)
9 (337–300 ka)	Schöningen	Orgnac 3 Cagny-L’Epinette	Bolomor Cave Gran Dolina TD10-1
10 (374 – 337 ka)			
11 (424–374 ka)	Clacton	Cueva del Angel (MIS 11–9) Terra Amata	
12 (478–424 ka)		La Micoque (MIS 12–11) Caune de l’Arago	
13 (533–478 ka)		Boxgrove	

The transition from the Acheulean to prepared core and Mousterian technologies is marked by an increased use and abundance of soft organic knapping tools (retouchers).

In the Levant, early use of soft knapping hammers has been proposed for Gesher Benot Ya‘aqov (GBY) in Israel (~ 730–800 ka). A technological analysis of the lithic assemblage from GBY suggests that soft percussors were used to manufacture handaxes^80^. Although no convincing bone or wood knapping tools have been identified at GBY, Goren-Inbar^81^ illustrates a modified cervid antler that is interpreted as a possible knapping hammer; details of this intriguing find have yet to be fully published.

The earliest unambiguous use of bone and antler knapping tools is from Boxgrove, UK dating to about 500–480 ka. The site has yielded one of the largest assemblages of bone knapping tools together with the earliest examples of antlers modified for use as knapping hammers^27,56,60,82,83^. The associated flint handaxes display advanced workmanship with tips sharpened with tranchet removals, straight edges, and a high degree of planform symmetry. The British Acheulean record also includes assemblages of roughly made and irregularly-shaped handaxes knapped with a few hard-hammer removals. These are exemplified by the assemblages from Fordwich^84^ and the Breccia at Kent’s Cavern^85^. The two groups of finely-shaped handaxes on the one hand and the crudely worked handaxes on the other exemplifies a major typological division, which is probably determined solely by the types of percussor(s) used in their manufacture.

The European Lower Palaeolithic site with the largest assemblage of organic percussion tools is, however, associated with the ~ 300 ka core-and-flake industry at Schöningen, Germany^17,18,86^. Here, the lithic assemblage consists of denticulates, notched pieces, scrapers and points and small chips from the intensive resharpening of the flake tools^87^ at butchery sites. The intensive re-sharpening of the flint tools using bone retouchers appears to reflect the difficulty of procuring flint raw material and the absence of pebbles suitable for hammerstones at this waterside location. The use of bone hammers replacing hammerstones for knapping and marrow extraction tasks is a behaviour that is unique to Schöningen^23^. This situation contrasts with the Clacton riparian locations where flint gravel provided an ample supply of cobbles suitable for use as hammerstones. The use of long bones for knapping in this context suggests that these bones were chosen for practical reasons relating to their specific properties as knapping tools.

## Conclusion

The bone tools from Clacton identified in this study provide a rare glimpse at the use of organic tools associated with a technologically ‘simple’ Lower Palaeolithic core-and-flake industry some 400,000 years ago. These tools are interpreted as knapping hammers, providing a link with processes used to manufacture Clactonian lithic tools. They represent the earliest known use of organic soft hammers associated with a Mode 1 lithic industry and they challenge the view that Clactonian flintknapping was undertaken solely with hard hammerstones. The bone hammers document a previously unexpected aspect of Clactonian behavioural complexity, which shows that this group was applying new raw materials to tasks that were formerly confined to the lithic realm. Further re-examination of faunal remains from other historic collections would likely reveal further bone tools that would contribute to a more nuanced understanding of the importance of non-lithic raw materials in the Lower Palaeolithic.

The relatively simple Clactonian knapping technology has been used to infer less sophisticated cognitive and behavioural abilities relative to other hominin groups with handaxe technology. However, this interpretation should be tempered in the knowledge that the non-lithic component of Mode 1 technologies at Clacton and Schöningen included specialized wooden projectile weapons (spears, throwing sticks) and thrusting spears (which are still unknown from any Acheulean context)^28,89,90^ coupled with evidence from Clacton for hunting of prime-aged rhinoceros (unpublished data). By contrast, handaxes at many Lower Palaeolithic sites, such as Fordwich^84^, Kent's Cavern^85^, the Bytham River in the Brecklands (UK)^91^ and Abbeville (France)^92^, were produced solely with hard hammers, reflecting a technologically simpler Mode 2 knapping technology.

The simple division of the Lower Palaeolithic into Mode 1 and Mode 2 technologies, governed by a bias towards lithic evidence, overshadows other types of material culture and the glimpses of complex behaviour that they occasionally reveal. It begs the question of the utility of the mode system, other than as a shorthand for lithic assemblage description. Evidence that also identifies non-lithic technologies, proficiency in hunting and butchery, structured and extended landscape use, alongside developed social behaviour, is more likely to map on to the complexity of human lineages that is increasingly being revealed through studies of hominin fossils, ancient DNA and proteonomics^93–95^.

## Supplementary Information

Supplementary Information.

## Data Availability

The datasets generated during the current study are available from the corresponding author on reasonable request.
