# Supplementary material for: Taphonomic and technological analyses of Lower Palaeolithic bone tools from Clacton-on-Sea, UK
Source: Sci Rep. 2022 Nov 23;12:20222. doi: 10.1038/s41598-022-23989-x (PMC9684524; doi:10.1038/s41598-022-23989-x)
Supplement: Supplementary file 1 — Supplementary Information. [file 41598_2022_23989_MOESM1_ESM.pdf]

Supplementary Information

Taphonomic and technological analyses of Lower Palaeolithic bone tools from Clacton-on-Sea, UK

Simon A. Parfitt, Mark D. Lewis & Silvia M. Bello

Pollen analysis

Methods

Sediment samples removed from the medullary cavity of the red deer tibia (NHMUK PA E 7492**)** and adhering to the surface of the rhinoceros radius (NHMUK PV M 103081**)** were prepared for pollen analysis by standard procedures to remove carbonates, silicates and particles >150 microns. Samples were stained using safranin and mounted in glycerine jelly. Counting was done by equally-spaced traverses across slides at 400x magnification with phase contrast, oil immersion (1000x) and occasionally laser confocal microscopy used for more problematic identifications. A minimum of 400 land pollen and spores was counted. Pollen identifications were made using the key of Moore *et al.*[1]; reference collections were also consulted. Pollen nomenclature follows that of Moore *et al.*[1] and plant nomenclature that of Stace[2]. Indeterminable grains were recorded as an indication of the state of pollen preservation.

The results are presented with taxa expressed as percentages of the total land pollen and spore sum (calculation sum). Obligate aquatic taxa and other palynomorphs are presented as percentages of the calculation sum + the sum of the category to which they belong. Both samples contained countable pollen, although pollen preservation from both was poor, with degradation and corrosion resulting in indeterminable grains, and others concealed, broken, folded or crumpled.

Results

The pollen spectrum from the radius (NHMUK PV M 103081: Table S1) is characterized by high values of *Quercus* and *Alnus*, and low values of *Corylus*. *Betula* and *Ulmus* are well represented at about 11% and 5% TLP respectively, and there are single grains of *Acer* and *Taxus*. *Pinus* values are also high and *Picea* is present. However, *Abies*, characteristic of the Hoxnian from zone Ho III onwards is absent. *Hedera* and *Ilex* are represented, as is Type X (4.4%. Fig. 2b), a tricolpate grain still of uncertain affinity but characteristic of the Hoxnian interglacial, although Type X pollen has also now been identified in MIS 9 deposits at Cudmore Grove[3]. Herbaceous taxa are relatively common, including Poaceae (11%), and together with the presence of aquatic plant taxa and three freshwater algal taxa suggest a spectrum characteristic of fluviatile temperate interglacial conditions. This is supported by the presence of twenty-two dinoflagellate cyst taxa, identified by Dr Geoff Eaton as mainly Eocene in age and most likely reworked from bedrock within the river catchment (Geoff Eaton, pers. comm.). A tentative vegetational interpretation from this one spectrum would be of a river valley, possibly with backwaters, and nearby grassland and mixed deciduous forest.

Specimen	NHMUK PV M 103081		NHMUK PA E 7492	
Sample weight (g)	1.8		3.0	
Count	n	%	n	%
**Trees and shrubs**				
Acer	1	0.2	-	-
Alnus	87	21.4	2	0.3
Betula	43	10.6	3	0.5
Picea	8	2.0	-	-
Pinus	59	14.5	19	3.0
Quercus	55	13.5	17	2.7
Taxus	1	0.2	-	-
Tilia	2	0.5	16	2.6
Ulmus	20	4.9	2	0.3
Corylus	14	3.4	4	0.6
Hedera	17	4.2	1	0.2
Ilex	2	0.5	-	-
Type X	9	2.2	-	-
cf. Type X	9	2.2	-	-
Ericaceae undiff.	3	0.7	3	0.5
**Herbs**				
Poaceae	44	10.8	130	20.8
Cyperaceae	4	1.0	5	0.8
Armeria maritima	1	0.2	7	1.1
Asteraceae undiff.	1	0.2	10	1.6
Artemisia	-	-	1	0.2
Aster-type	2	0.5	2	0.3
Cirsium-type	-	-	1	0.2
Brassicaceae	-	-	2	0.3
Caryophyllaceae	-	-	17	2.7
Chenopodiaceae	1	0.2	285	45.6
Persicaria maculosa	1	0.2	-	-
Plantago undiff.	1	0.2	45	7.2
Polygonum cf. *aviculare*-type	1	0.2	1	0.2
Ranunculus-type	4	1.0	1	0.2
Rosaceae undiff.	-	-	1	0.2
cf. *Alchemilla*	-	-	1	0.2
cf. *Filipendula*	-	-	1	0.2
Rumex acetosa-type	3	0.7	2	0.3
Taraxacum-type	1	0.2	19	3.0
Thalictrum	-	-	1	0.2
Apiaceae	-	-	1	0.2
Urtica	2	0.5	-	-
**Pteridophytes**				
Dryopteris-type	1	0.2	-	-
Equisetum	1	0.2	-	-
Polypodium	1	0.2	18	2.9
Pteridium	1	0.2	-	-
Pteropsida monolete undiff.	7	1.7	7	1.1
**Calculation sum (total land pollen & spores)**	407	100.0	625	100.0
**Aquatics**				
Alisma-type	1	0.2	-	-
Myriophyllum spicatum	1	0.2	-	-
Potamogeton	1	0.2	-	-
Sparganium/Typha angustifolia-type	3	0.7	3	0.48
**Freshwater algae**				
Botryococcus	4	0.9	-	-
Pediastrum	5	1.2	-	-
Zygnema-type	9	2.1	-	-
**Other palynomorph categories**				
Sphagnum	-	-	1	0.16
Trilete undiff.	1	0.2	7	1.1
Dinoflagellata undiff.	31	7.1	-	-
Indeterminate	44	9.8	25	3.85
Unknown	-	-	4	0.64

Table S1. Counts and percentages of the pollen spectra from sediment adhering to the rhinoceros radius (NHMUK PV M 103081) and red deer tibia (NHMUK PA E 7492).

The spectrum shows clear affinities with British Hoxnian interglacial pollen assemblages but appears to be earlier in the interglacial succession than those of the freshwater sediments at the base of Borehole A of Pike and Godwin[4], which show a clear presence of *Abies* and *Carpinus*, and are considered to belong to subzone Ho IIIa. However, it can be compared with spectra from Borehole B[5], and more importantly with the more informative pollen sequence from Marks Tey[6].

In comparison with the two pollen spectra from Borehole B there are similarities in the assemblages, such as the high values for *Quercus* and *Alnus*, low values for *Corylus*, as well as the presence of *Hedera*, *Ilex* and Type X and similar Poaceae values. However, unlike Borehole B, the spectrum from the rhinoceros radius differs in having a higher *Ulmus* value, at about 5%, and the presence of *Acer* and *Taxus*. Additionally, there are differences in the relative frequencies of both *Betula* and Type X. It is suggested that the spectra from Borehole B belong to subzone Ho IIb[5]. Undoubtedly, the similarities allow us to place the radius in an interglacial period but the differences here are also noticeable, and comparison with a more complete sequence is needed.

Marks Tey provides the most comprehensive vegetational history for the Hoxnian interglacial in Britain and can potentially help to establish the context of the rhinoceros radius. Although the relative values of *Quercus* and *Alnus* are slightly lower than those seen in Ho IIb of Turner[6] at Marks Tey, they are still the dominant deciduous taxa, and other floristic elements of the spectrum of this specimen, such as the near absence of *Taxus*, cannot be ignored. Bridgland *et al.*[7], assign one of their sequences at Clacton (Butlin's holiday camp section F6.4) to Ho IIc when compared with Turner[6], high frequencies of *Taxus* clearly being characteristic of this subzone. However, the spectrum of the sample from the radius represents only one sample and any interpretation, further hindered by a lack of detailed lithology, should be treated with caution. Additionally, local vegetational differences should be borne in mind. Based on the observations above, including the occurrence of the palynomorph Type X, low *Corylus* values, and the presence of *Ilex*, scarce *Taxus* and absence of *Abies*, the pollen spectrum from the rhinoceros radius can probably be assigned to subzone Ho IIb.

The red deer tibia was excavated from deposits on the foreshore at Jaywick. The stratigraphy of this area is more complex with at least three distinct channels recorded by Warren: the main Hoxnian channel (v); Channel iv to the east, which is now known to be Last Interglacial in age[8,9]; and to the west there is another post-Hoxnian channel ('vi'). The post-Hoxnian channels contain estuarine deposits, but they incorporate earlier stone tools and bones reworked from the Hoxnian Channel. A further confounding factor is the presence of Holocene estuarine, alluvial and terrestrial deposits that mantle the Pleistocene sediments and extend onto the foreshore at Jaywick. These deposits also contain reworked Pleistocene material. We consider the tibia to have been reworked from Hoxnian sediments, a conclusion which is supported by the two phases of weathering and abrasion. These features, and the information provided by the pollen analysis, are consistent with the bone having been reworked and redeposited into Holocene sediments.

The pollen spectrum from sediment extracted from the medullary cavity of the deer tibia (NHMUK PA E 7492: Table S1) cannot be matched to any level of either Boreholes A

or B of Pike and Godwin[4] or Turner and Kerney[5]; it does, however, show strong affinity to spectra from the Holocene deposits in Section C at the Butlin's site[7]. Although the pollen spectrum is interglacial in character, tree values are very low, with only *Pinus, Quercus* and *Tilia* each reaching about 3% total land pollen (TLP; Table S1). The spectrum is heavily dominated by Chenopodiaceae (45%), which with Poaceae and *Plantago* account for about 73% TLP. *Armeria maritima* is also present, and together with the high Chenopodiaceae numbers, probably represents saltmarsh conditions. There is a large diversity of herb taxa, although the only noteworthy values are of Caryophyllaceae, and Asteraceae and *Taraxacum*-type (equivalent to the Compositae Tubuliflorae and Liguliflorae of Bridgland *et al.*[7]). A small number of observed Poaceae grains >40µ diameter may be of cereal type. *Polypodium* is also present. The sediment from the bone cavity represents only a single sample, so any interpretation should be treated very cautiously. With that proviso, and particularly from the high Chenopodiaceae counts, pollen from the tibia shows a closer affinity with the Holocene pollen spectra of Section C[7]. Further palynological work on sequences in the Jaywick area may help to locate the context of this specimen more closely.

References

1. Moore P.D., Webb J.A. & Collinson M.E. *Pollen Analysis*. (Blackwell Scientific Publications, 1991).

2. Stace, C. *New Flora of the British Isles.* (Cambridge University Press, 2010).

3. Roe, H.M., *et al.* Differentiation of MIS 9 and MIS 11 in the continental record: Vegetational, faunal, aminostratigraphic and sea-level evidence from coastal sites in Essex, UK. *Quat. Sci. Rev.* **28,** 2342–2373 (2009).

4. Pike, K. & Godwin, H. The interglacial at Clacton-on-Sea, Essex. *Quart. J. Geol. Soc.* **108,** 261–72 (1953).

5. Turner, C. & Kerney, M.P. A note on the age of the freshwater beds of the Clacton Channel. *J. Geol. Soc.* **127,** 87–93 (1971).

6. Turner, C. The Middle Pleistocene deposits at Marks Tey, Essex. *Philos. Trans. R. Soc. London* **B257,** 373–440 (1970).

7. Bridgland, D.R., *et al.* Middle Pleistocene interglacial Thames–Medway deposits at Clacton-on-Sea, England: Reconsideration of the biostratigraphical and environmental context of the type Clactonian Palaeolithic industry. *Quat. Sci. Rev.* **18,** 109–146 (1999).

8. Allen, P. *et al*. Mid-Late Quaternary fluvial archives near the margin of the MIS 12 glaciation in southern East Anglia, UK: Amalgamation of multi-disciplinary and citizen-science data sources. *Quaternary* **5, 37,** https://doi.org/10.3390/quat5030037 (2022).

9. Penkman, K.E.H. *et al.* An aminostratigraphy for the British Quaternary based on *Bithynia* opercula. *Quat. Sci. Rev.* **61,** 111–134. (2013).
